# Role of inflammation in neurological damage and regeneration following spinal cord injury and its therapeutic implications

**DOI:** 10.1093/burnst/tkac054

**Published:** 2023-02-28

**Authors:** Yan Jin, Yixing Song, Jiaqi Lin, Tianqing Liu, Guicai Li, Biqin Lai, Yun Gu, Gang Chen, Lingyan Xing

**Affiliations:** Key Laboratory of Neuroregeneration of Jiangsu and the Ministry of Education, Co-innovation Center of Neuroregeneration, NMPA Key Laboratory for Research and Evaluation of Tissue Engineering Technology Products，Nantong University, Nantong 226006, China; School of Life Sciences, Nantong University, Nantong 226019, China; Key Laboratory of Neuroregeneration of Jiangsu and the Ministry of Education, Co-innovation Center of Neuroregeneration, NMPA Key Laboratory for Research and Evaluation of Tissue Engineering Technology Products，Nantong University, Nantong 226006, China; School of Medicine, Nantong University, Nantong 226006, China; NICM Health Research Institute, Western Sydney University, Westmead, NSW 2145, Australia; Key Laboratory of Neuroregeneration of Jiangsu and the Ministry of Education, Co-innovation Center of Neuroregeneration, NMPA Key Laboratory for Research and Evaluation of Tissue Engineering Technology Products，Nantong University, Nantong 226006, China; Key Laboratory for Stem Cells and Tissue Engineering (Sun Yat-sen University), Ministry of Education, Guangzhou 510275, China; Co-innovation Center of Neuroregeneration, Nantong University, Nantong 226006, China; Key Laboratory of Neuroregeneration of Jiangsu and the Ministry of Education, Co-innovation Center of Neuroregeneration, NMPA Key Laboratory for Research and Evaluation of Tissue Engineering Technology Products，Nantong University, Nantong 226006, China; School of Medicine, Nantong University, Nantong 226006, China; Key Laboratory of Neuroregeneration of Jiangsu and the Ministry of Education, Co-innovation Center of Neuroregeneration, NMPA Key Laboratory for Research and Evaluation of Tissue Engineering Technology Products，Nantong University, Nantong 226006, China

**Keywords:** Inflammation, Immune response, Spinal cord injury, Axon regeneration, Cell death, Zebrafish, Inflammatory drugs, Trauma, Neurological damage

## Abstract

Spinal cord injury (SCI) is an incurable trauma that frequently results in partial or complete loss of motor and sensory function. Massive neurons are damaged after the initial mechanical insult. Secondary injuries, which are triggered by immunological and inflammatory responses, also result in neuronal loss and axon retraction. This results in defects in the neural circuit and a deficiency in the processing of information. Although inflammatory responses are necessary for spinal cord recovery, conflicting evidence of their contributions to specific biological processes have made it difficult to define the specific role of inflammation in SCI. This review summarizes our understanding of the complex role of inflammation in neural circuit events following SCI, such as cell death, axon regeneration and neural remodeling. We also review the drugs that regulate immune responses and inflammation in the treatment of SCI and discuss the roles of these drugs in the modulation of neural circuits. Finally, we provide evidence about the critical role of inflammation in facilitating spinal cord neural circuit regeneration in zebrafish, an animal model with robust regenerative capacity, to provide insights into the regeneration of the mammalian central nervous system.

Highlights

- The complex roles of inflammation in neural circuit events following SCI are discussed.
- The drugs regulating immune responses and inflammation in the treatment of SCI are reviewed, as well as their roles in the pathophysiology of SCI.
- The positive roles of inflammation in facilitating spinal cord neural circuit regeneration in zebrafish are reviewed.

## Background

The inflammatory responses that occur after spinal cord injury (SCI) are complicated and can have both beneficial and detrimental effects. For example, drugs that suppress immune responses or inflammation, such as methylprednisolone and minocycline, have been employed to treat SCI [1,2]. On the other hand, drugs that activate inflammatory signaling can also mitigate secondary damage [3]. The use of these medications is constrained by their poor therapeutic efficacy and the possibility of negative side effects. To date, no effective anti-inflammation drugs have been developed for SCI, primarily due to limited understanding of the SCI-associated inflammatory responses. For instance, researchers are still looking into the periods in which inflammation plays a role as well as the biological processes that inflammation affects. Poor functional recovery in SCI is a result of the degeneration of neural circuits brought on by neuronal loss, axonal retraction or synaptic damage, which limits the processing of ascending or descending signals. Therefore, it is crucial to comprehend how inflammation affects modifications to neural circuits following SCI.

While mammalian SCI is irreversible, some teleosts, including zebrafish, have a high capacity for neural circuit regeneration [4]. Because inflammation develops differently in fish and mammals, this divergence could lead to a significant advance in the research of regeneration.

## Review

### Immune and inflammation responses in SCI

**Figure 1:**
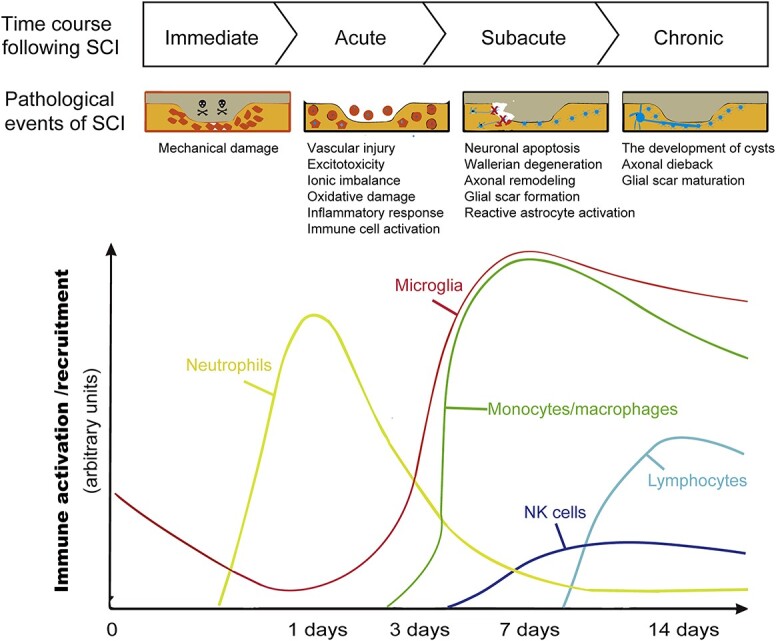
Cellular events following SCI over time. Damage and cellular events, as well as immune cell activation, are revealed. Primary injury triggers a pathophysiological cascade, including the secondary injury stage. Secondary injury begins as early as a few minutes after the initial trauma and is divided into three stages: acute, subacute and chronic injury. The changes of various cells (neutrophils, microglia, monocytes/macrophages, lymphocytes and NK cells) in the process of SCI are marked. This Figure is modified from [11,99] with permission. *SCI* spinal cord injury, *NK cells *natural killer cells

SCI consists of primary injury and secondary injury. Primary injury is related to the initial traumatic injury and is caused by the destructive impact, resulting in immediate and irreversible mechanical injury. Primary injury triggers a pathophysiological cascade, including the secondary injury stage. Secondary injury begins as early as a few minutes after the initial trauma and is divided into three stages: acute, subacute and chronic [5–8]. Although the pathological mechanism of SCI is similar across different species, the rate of illness evolution varies, such as between mice and humans. In rodents, secondary injury can be divided into an acute stage (<24 h), a subacute stage (from 24 h to 7 days) and a chronic stage (>7 days) [8]. In humans, the transition between the acute phase and subacute phase usually occurs within a few hours to 48 h after injury, and the change from acute to chronic phase is thought to happen at 6 months [5]. The acute secondary injury stage is characterized by vascular injury, excitotoxicity, ionic imbalance, oxidative damage, the inflammatory response and immune cell activation. Acute secondary injury leads to the subacute secondary injury stage, which is characterized by neuronal apoptosis, Wallerian degeneration, reactive astrocyte activation, axonal remodeling and glial scar formation. Subacute secondary injury leads to chronic secondary injury, which is characterized by the development of cysts, axonal dieback and glial scar maturation (Figure 1) [6,7,9,10].

Immediately after SCI, neutrophils and microglia become activated and secrete a number of cytokines, such as interleukin-1β (IL-1β), tumor necrosis factor alpha (TNF-α) and interleukin 6 (IL-6). This causes the subsequent infiltration of monocyte-derived macrophages, which play an important role in scavenging tissue debris at the lesion site. During the subacute phase, monocytes and macrophages infiltrate the spinal cord and produce proinflammatory cytokines, chemokines, autoantibodies, reactive oxygen and nitrogen species and other inflammatory mediators. The neuroinflammatory response is important for secondary injury, which results in cell death and tissue degeneration in the subacute and chronic stages. The inflammatory process and secreted cytokines have been extensively examined [11]. In this review, we will concentrate on the role of inflammation in different damaged neural circuits after SCI.

### Cell death and inflammation

There are two main types of cell death, non-programmed cell death and programmed cell death. Non-programmed cell death, also known as necrosis, is an unregulated passive process. It is generally accepted that this type of non-programmed cell death can be triggered by proinflammatory cytokines, glutamate excitotoxicity, ionic imbalance and toxic components. Apoptosis, necroptosis, autophagy, ferroptosis and pyroptosis are examples of programmed cell death, which is a type of cell death controlled by regulatory mechanisms. Pyroptosis is a form of cell death that is triggered by proinflammatory signaling and requires the activation of caspase-1 and/or caspase11 (caspase 4/5 in humans). Pyroptosis is most commonly observed in phagocytes of the myeloid lineage, such as macrophages, dendritic cells and neutrophils [12]. The function of inflammasomes in pyroptosis has been thoroughly covered in other articles [13]. Here we will focus on apoptosis and necroptosis.

### Apoptosis and inflammation

Apoptosis has been considered to be an immunologically silent form of cell death, and dying cells are cleared by phagocytes during this process. Apoptosis does not result in a proinflammatory reaction because the membrane of dying cells that are engulfed by phagocytes is still intact and the cell contents do not come into contact other surrounding cells [14]. Furthermore, apoptotic cells maintain this anti-inflammatory state even after entering secondary necrosis and leaking their cellular contents [14,15]. Apoptosis can be regulated by inflammation even when it does not trigger proinflammatory responses. TNF-α is a proinflammatory cytokine that can induce apoptosis in non-immune cells via its receptor TNF receptor 1, whose intracellular death domain activates caspase-8 and caspase-10, resulting in cell death. This process can be regulated by other inflammatory signaling pathways. For example, inducible NO synthase is a key inflammatory mediator that can activate TNF-α-mediated signaling [16,17]. Fas (also known as CD95) is another extrinsic immune-related signaling protein that is expressed by multiple cell types after SCI, including astrocytes, oligodendrocytes and microglia. In both the acute and subacute stages of SCI, activation of this cell-death receptor causes apoptosis and an inflammatory response [18]. Following SCI, Fas-deficient mice have a significant decrease in apoptosis, as well as inflammation, as shown by a decrease in cytokine expression [18]. Although cell death is unavoidable during primary SCI, studies have shown that inhibiting apoptosis can promote functional recovery. Interestingly, inhibiting Fas signaling in transgenic fas-deficient mice or administering soluble Fas receptor to block receptor activity can significantly improve functional recovery after SCI [18,19].

Other evidence suggests a correlation between inflammation and apoptosis. For example, an increase in p38 expression was associated with inflammation and apoptosis after SCI, and the p38 inhibitor SB203580 alleviated secondary SCI by suppressing inflammation and apoptosis [20]. Deficiency in progranulin, a 593 amino acid-long secreted glycoprotein, is detrimental to SCI recovery by promoting apoptosis and neuroinflammation [21]. Metformin inhibited neuronal apoptosis and the inflammatory response by increasing the expression of catenin and brain-derived neurotrophic factor and promoted motor functional recovery in rats following SCI in a recent study [22]. The above research results show that studying the relationship between apoptosis and inflammation and looking for ways to inhibit apoptosis after SCI may have important clinical significance for further treatment of SCI.

### Necroptosis and inflammation

Necroptosis, which is also known as programmed necrosis, results in similar morphological changes to necrosis, such as ruptured cell membranes, enlarged cell volumes and swollen organelles. In necroptosis, activated TNF-α stimulates receptor-interacting protein kinases 1/3 (RIPK1/3) and mixed lineage kinase domain-like protein (MLKL) signaling. In contrast to apoptosis, necroptosis requires RIPK activity, which is regulated by a caspase-independent pathway [23]. After SCI, the expression levels of RIPK1, RIPK3 and MLKL are upregulated, although their peak expression varies temporally; RIPK3 and MLKL peak 1 day post-injury (dpi) [24] and RIPK1 peaks 3 dpi [25]. Inhibiting MLKL activity in SCI can facilitate neurological function recovery [26].

It has been discovered that multiple inflammatory signaling pathways are actively involved in the process of necroptosis. Reactive astrocytes undergo necroptosis in response to the inflammatory response-related gene toll-like receptor 4 (TLR4) and myeloid differentiation primary response gene 88 [27,28]. Lipopolysaccharide-induced neuroinflammation promotes necroptosis in neurons, which is facilitated by smad ubiquitination regulatory factor 1 [29]. According to a study showing that the necroptosis inhibitor necrostatin-1 can lessen tissue loss and speed up functional recovery following SCI in mice, suppressing necroptosis appears to be advantageous [30]. Notably, in contrast to apoptosis, necroptosis results in the release of cytokines or other inflammatory mediators into the extracellular environment. Therefore, there might be complex crosstalk between inflammation and necroptosis.

Future investigations should look into the relationship between inflammation and cell death, specifically programmed cell death, in secondary injury in SCI. It would also be intriguing to investigate whether cell death is a potential target for inflammation-modulated SCI therapy.

### Role of inflammation in neural remodeling and axon regeneration

Both neural remodeling and axon regeneration are essential biological processes for the reorganization of neural circuitry, and inflammation signaling appears to play multiple roles in both of these processes. Microglia can interact with damaged axons and phagocytose dendrites [31], potentially exacerbating synaptic damage. This process may involve matrix metallopeptidase 9 and chemokine [32–34]. Axon sprouting, synaptic remodeling and functional recovery are all enhanced by deletion of CX3CR1, a chemokine receptor that binds to CX3CL1 in microglia/macrophages [35]. On the other hand, inflammatory stimulation can also promote axon regeneration after SCI. Intraspinal injection of the TLR2 agonist Pam2CSK4 prevents axonal dieback [3]. This outcome is consistent with research that indicates that the potent inflammatory stimulus zymosan can encourage axon regeneration by activating macrophages [36].

It is worth noting that macrophages can either promote axon regrowth or exacerbate tissue remodeling. The different roles of macrophages may come from their heterogeneity. Many of the negative effects of macrophage interactions have been attributed to the M1-like phenotype, which orchestrates glutamate- and nitric oxide-induced neuronal death [37]. M2-like macrophages, which are normally repressed, are beneficial, and their activation may reduce axonal dieback by reducing gliosis and the expression of proinflammatory cytokines such as IL-1 [38,39]. Therefore, when targeting macrophages, the diverse roles of macrophages must be considered [36].

Other immune-related cells associated with axonal recovery have been identified. Neutrophils are a diverse immune cell population that is essential for immune defense. A subset of neutrophils with axon regenerative properties has been discovered [40], which is defined as CD14 + Ly6Glo granulocytes, and these cells resemble immature neutrophils. This subpopulation promotes axonal regrowth in SCI [40]. In SCI, activated B cells secrete the antibody complement component 1q, which exacerbates axon pathology and demyelination, and this effect is ameliorated in mice with B-cell deficiency [41]. Leukocytes can be induced by IL-6 or leukemia inhibitory factor and inhibit axonal growth and impair locomotor recovery [42]. T cells can be activated by vaccination with myelin basic protein and exacerbate demyelination and axonal pathology in both mice and rats, leading to increased tissue destruction [43,44]. Inflammatory signals generated or stimulated by these cells may be critical for functional recovery after SCI. Therefore, it is necessary to investigate how various cell types orchestrate inflammatory signaling and contribute to neural remodeling.

### Zebrafish inflammation in SCI

Both larval and adult zebrafish can regenerate axons and functionally recover from SCI [45]. Because of the high molecular and genetic conservation between teleosts and mammals, zebrafish research may be able to overcome the limitations of non-regenerative mammalian models.

Zebrafish with central nervous system injury may benefit from acute inflammation, which promotes healing [46]. For instance, injection of the inflammatory mediator IL-6 into the ventricles of embryonic zebrafish promotes spinal cord axon regrowth [47]. Microglia/macrophages in zebrafish express typical vertebrate macrophage genes, including a variety of transcriptional regulators, immune pathogen receptors and pruning-associated genes, suggesting functional conservation between mammals and fish [48]. It is interesting to note that in early SCI, many M2-type macrophage genes are upregulated, whereas changes in the expression of M1-type macrophage genes are very limited [49]. This rapid expansion of M2 anti-inflammatory macrophages may contribute to the advantageous benefits of acute inflammation in zebrafish.

**Table 1:** Popular drugs targeting inflammation in the treatment of SCI

**Drugs**		**Inflammation processes regulated**	**Biological processes affected**	**Beneficial or detrimental for function recovery**	**Reference**
PPAR-γ agonists	15d-PGJ_2_	15d-PGJ_2_ prevents neutrophil infiltration	Reduces cell death in neurons and oligodendrocytes	Improves motor, sensory function and neuroprotection	[64–67]
	Rosiglitazone	Rosiglitazone exerts anti-inflammatory effects by inhibiting NLRP3 inflammasome in neurons			
	Pioglitazone	Pioglitazone reduces inflammatory genes			
Methylprednisolone		Binds with glucocorticosteroid receptors to prevent nuclear translocation of proinflammatory transcription factors	Reduces astrocyte cell death and microglial activation, inhibits A1 astrocytes activation and free oxygen radical induced lipid peroxidation	Improves motor and sensory function but is still controversial in clinical practice Increases the extravasation of plasma components and enhances tissue swelling and edema High dose administration can lead to a variety of physical complications, including wound infection, sepsis, gastrointestinal bleeding, pulmonary embolism and even mortality	[1,68–71]
Minocycline		Inhibits the proinflammatory and neurotoxic cytokines TNF-α and IL-1β and reduces microglial/macrophage activation	Reduces the lesion area, increases the number of descending sympathetic axons passing through the injury site, inhibits inflammation, axonal dieback and microglial/macrophage activation and improves axonal regeneration	Improves motor function and has neuroprotective effect	[2,71–75]
Erythropoietin		Inhibits leukocytes infiltration and reduces the level of pro-inflammatory cytokines such as TNF-α, IL-6 and MCP-1	Prevents neuronal apoptosis through crosstalk between JAK-2 and NF-κB signal cascade Promotes neurogenesis and oligodendrocyte survival, enhances axon regeneration and reduces myelin loss through activating AMPK and inactivating mTOR signals	Improves motor function and has neuroprotective effect Long-term administration can lead to excessive erythropoiesis and increased blood viscosity.	[76–79]
Estrogen		Inhibits the activation of microglia and astrocytes	Reduces edema and the inflammatory response, improves axonal and myelin loss, and decreases cell death	Improves motor and sensory function and relieves neuropathic pain, but is still controversial in clinical practice High-dose administration has serious safety problems, such as the risk of deep venous thrombosis and coronary heart disease	[70,77,80,81]
Etanercept (anti-TNF-α agents)		Inhibites TNF-α and IL-1β expression	Modulates the post-traumatic inflammatory response, attenuates neuronal injury, reduces tissue damage and cell apoptosis	Improves motor function and reduces mechanical allodynia	[70,82,83]
Rolipram (PDE4 inhibitor)		Inhibits TNF-α and IL-1β production, prevents IL-10 reduction and increases white matter sparing	Protects neurons and oligodendrocytes, promotes axon regeneration and attenuates the formation of glial scars	Improves motor function, promotes neuroprotection and enhances myelinated tissue sparing Side effects such as nausea, vomiting and sedation may occur during use	[70,84–89]
Anti-α4β1 integrin		Decreases the intraspinal influx of neutrophils and monocyte/macrophages and reduces leukocyte activation and migration	Inhibits inflammation and secondary injury	Improves motor function, decreases neuropathic pain and has neuroprotective effect	[70,90,91]
Substance P		Stimulates IL-10 expression and induces M2 macrophages	Activates neural stem cells, increases neuronal cells and reduces apoptotic cells	Improves motor function and repairs damaged tissue	[92–95]
G-CSF		Alleviates inflammation signaling and promotes M2 macrophage activation	Inhibits the apoptosis of nerve cells and oligodendrocytes, reduces glial scars, enhances axonal myelination and regeneration	Improves motor and sensory function Side effects such as urinary tract infection, mild hepatopathy and gastric ulcer in clinical trials	[62,69,71,96–98]

*SCI* spinal cord injury, *PPAR* peroxisome proliferator-activated receptor, *15d-PGJ_2_* 15-deoxy-Δ^12,14^-PGJ_2_, *NLRP3* NLR family pyrin domain containing 3, *TNF-α* tumor necrosis factor α, *IL-1β* interleukin-1β, *IL-6* interleukin-6, *MCP-1* macrophage chemotactic protein-1, *JAK* janus kinase, *NF-κB* nuclear factor kappa-light-chain-enhancer of activated B cells, *AMPK* 5′adenosine monophosphate-activated protein kinase, *mTOR* mammalian target of rapamycin, *PDE4* phosphodiesterase 4, *IL-10* interleukin-10, *G-CSF* granulocyte-colony stimulating factor

Rapid remission of inflammation is observed in zebrafish following SCI. Temporal activation of microglia and macrophages differs between zebrafish and mammals, although early activation of microglia and the infiltration of blood-borne macrophages have been observed at the wound site in the two species [50]. In mammals, macrophages and microglia persist at the injury site (for at least 42 days in rodents and 12 months in humans) [51–53]. After damage, M1-polarized macrophages persist for an extended period of time and exert neurotoxic effects that result in chronic inflammation and poor functional recovery [54–56]. However, macrophage depletion was observed 10 dpi in adult zebrafish spinal cords [50], while in larval zebrafish, microglia and macrophages peaked at 2 dpi before significantly declining by 5 dpi [47]. Additionally, neutrophil counts peaked at 2 hour post injury in larval zebrafish and then rapidly declined thereafter [47]. The rapid resolution of inflammation in zebrafish SCI may provide a favorable extracellular environment for axon growth.

Regulatory T cells, a distinct subtype of T cells that plays a central role in preserving self-antigen tolerance and reducing inflammatory tissue injury, are another element in the treatment of SCI. Zebrafish regulatory T cells quickly migrate to the injured region in damaged organs and produce tissue-specific regenerative factors through a procedure distinct from the classic anti-inflammatory pathway to promote the proliferation of regenerative precursor cells [57].

These findings provide compelling evidence that inflammation plays a role in the pathogenesis of SCI. The timing and level of immune cell activation may determine whether inflammation is beneficial or detrimental. The results in zebrafish show that acute inflammation is mostly advantageous. However, prolonged inflammation may deteriorate the reorganization of neural circuits. Understanding how to maximize the role of acute inflammation in neural regeneration and efficiently resolve inflammation is important for the development of regenerative medicines.

### Immune-targeting strategies for spinal cord regeneration

To date, methylprednisolone is the only pharmacological agent that has received clinical approval for the treatment of SCI, but the risks associated with corticosteroid treatment (e.g. gastrointestinal bleeding and wound infection) and limitations in functional recovery restrict its use [1]. Despite these drawbacks, it has been demonstrated that methylprednisolone is beneficial in suppressing inflammation, particularly microglial and macrophage activation, which aids in preventing tissue loss. Minocycline is a clinically available antibiotic with anti-inflammatory properties. Numerous animal models have revealed promising neuroprotective effects of minocycline. Minocycline pretreatment and treatment can significantly prevent production of the proinflammatory and neurotoxic cytokines TNF-α and IL-1β, thereby significantly reducing spinal cord tissue damage and functional and sensory complications after injury [2]. In clinical trials, minocycline can effectively suppress inflammation [58], and its administration tends to promote sensory and function outcomes, though no statistical significance has been observed [59]. The combination of minocycline and methylprednisolone is more effective than either drug alone. After SCI, combination therapy can effectively lower levels of lipid peroxidation as well as TNF-α and IL-6 levels, preventing the death of neuronal and glial cells [60].

A variety of targeting strategies for microglia and macrophages have been suggested to be effective. Pro-inflammatory M1 macrophages and anti-inflammatory M2 macrophages have been identified as a result of research into macrophage heterogeneity [54]. Overall, a strategy that boosts M2 activation or prevents the activation of M1 cells is advantageous for SCI recovery. For example, the administration of cytokines associated with M2 activation, including IL-4, IL-10 and IL-13, improves functional outcomes in SCI. Blocking M1 activation by blocking IL-6 or TNF-α facilitates tissue repair and functional recovery [61]. Minocycline can reduce macrophage activation (Table 1). Granulocyte-colony stimulating factor (G-CSF) and substance P can induce M2 macrophages (Table 1). These strategies have significantly enhanced neural circuit reorganization in SCI. Clinical trials revealed that G-CSF can slightly improve sensory and motor function in SCI patients, as shown by anti-inflammatory, myelin-protective and axon-regenerative effects [62], though its long-term effects require further investigation [63]. Table 1 also summarizes additional anti-inflammatory drugs used in the treatment of SCI and their potential roles in neural circuits.

## Conclusions

Inflammation plays a complex role in SCI injury and regeneration. Generally, early inflammatory events are critical for removing pathogens and cellular debris, as well as limiting the severity of acute injury. Excessive inflammatory responses after acute stages may impair axonal regeneration, neuronal regrowth and remyelination, resulting in severe neurological dysfunction. Notably, some inflammatory events, such as the activation of M2 macrophages, may be beneficial for regeneration. Inflammation management has emerged as one of the most important therapeutic strategies preventing apoptosis and oxidative damage, as well as promoting angiogenesis and neuronal regeneration. Future studies may focus on the precise function of distinct immune cells and the downstream signaling networks triggered by inflammation, as well as the ideal way to develop regenerative medicine strategies by effectively targeting inflammation. Due to the complexity of inflammation, it is feasible that concurrently targeting multiple immune cells or signaling may have synergistic effects that separate signaling cannot foresee.

**Abbreviations**
dpi: Days post-injury; G-CSF: Granulocyte-colony stimulating factor; IL-1β: Interleukin-1β; IL-4: Interleukin-4; IL-6: interleukin-6; IL-10: interleukin-10; IL-13: interleukin-13; MLKL: Mixed lineage kinase domain-like protein; RIPK1/3: Receptor-interacting protein kinases 1/3; SCI: Spinal cord injury; TLR2: Toll-like receptor 2; TLR4: Toll-like receptor 4; TNF-α: Tumor necrosis factor α.

## Funding

This work was supported by the National Key R&D Program of China (2022YFA1105900), the National Natural Science Foundation of China (81701127), the Nantong Science and Technology Foundation of China (JC2021058) and the Large Instruments Open Foundation of Nantong University (KFJN2231, KFJN2275). TL is supported by the National Health and Medical Research Council (NHMRC) Early Career Fellowship (Grant No. 1112258) and WSU Vice-Chancellor’s Senior Research Fellowship.

## Authors’ contributions

Conceptualization: LX and GC. Writing—original draft: YS, YJ, JL and LX. Writing—review and editing: YJ, TL, LX and GC. Analysis and collation of literature: GL, BL and YG. Supervision: LX and GC. Project administration: LX. All authors have read and approved the final manuscript.

## Conflicts of interest

The authors declare that they have no conflicts of interest.
